# Impact of automated pop-up alerts on simultaneous prescriptions of antimicrobial agents and metal cations

**DOI:** 10.1186/s40780-024-00377-3

**Published:** 2024-09-27

**Authors:** Takanori Matsumoto, Taichi Matsumoto, Chiyo Tsutsumi, Yoshiro Hadano

**Affiliations:** 1 Department of Pharmacy, St. Mary’s Hospital, 422 Tsubuku-Honmachi, Kurume, Fukuoka, 830-8543 Japan; 2 Basic Medical Research Unit, St. Mary’s Research Center, 422 Tsubuku-Honmachi, Kurume, Fukuoka, 830-8543 Japan; 3 Faculty of Nursing, St. Mary’s College, 422 Tsubuku-Honmachi, Kurume, Fukuoka, 830-8558 Japan; 4 Division of Infection Control and Prevention, Shimane University Hospital, 89-1 Enyacho, Izumo, Shimane 693-8501 Japan

**Keywords:** Chelation, Antimicrobial agents, Metal cations, Automated pop-up alerts

## Abstract

**Background:**

Antimicrobial agents (AMAs) are essential for treating infections. A part of AMAs chelate with metal cations (MCs), reducing their blood concentrations. That drug-drug interaction could lead to a reduction of therapeutic efficacy and the emergence of drug-resistant bacteria. However, prescriptions ordering concomitant intake (co-intake) of AMAs and MCs are frequently seen in clinical settings. A method for preventing such prescriptions is urgently needed.

**Methods:**

We implemented pop-up alerts in the hospital's ordering and pharmacy dispensation support system to notify the prescriptions ordering co-intake of AMAs and MCs for physicians and pharmacists, respectively. To assess the effectiveness of the pop-up alerts, we investigated the number of prescriptions ordering co-intake of AMAs and MCs and the number of pharmacist inquiries to prevent co-intake of AMAs and MCs before and after the implementation of pop-up alerts.

**Results:**

Before the implementation of pop-up alerts, 84.5% of prescriptions containing AMA and MCs ordered co-intake of AMAs and MCs. Implementing pop-up alerts time-dependently reduced the proportion of prescriptions ordering co-intake of AMAs and MCs to 43.8% and 29.5% one year and two years later, respectively. The reduction of tetracycline-containing prescriptions was mainly significant. Before the implementation of pop-up alerts, the proportion of prescriptions in which pharmacists prevented co-intake of AMAs and MCs was 3.4%. Implementing pop-up alerts time-dependently increased proportions of such prescriptions to 20.9% and 28.2% one year and two years later.

**Conclusion:**

Implementing pop-up alerts reduced prescriptions ordering co-intake of AMAs and MCs and accelerated pharmacists to prevent co-intake of AMAs and MCs. The implementation of dual pop-up alerts in the hospital's ordering and pharmacy dispensation support system could help prevent co-intake of AMAs and MCs.

**Supplementary Information:**

The online version contains supplementary material available at 10.1186/s40780-024-00377-3.

## Background

Antimicrobial agents (AMAs) play a pivotal role in treating infectious diseases. On the other hand, inappropriate use of AMAs could cause multidrug-resistant bacteria [1, 2]. Pharmacists explore efficient methods to reduce the improper use of AMAs through educational intervention [3], prescription recommendations based on pathogen identification [4], and multi-faceted intervention [5]. Oral AMAs such as Quinolones, Tetracyclines, and Cefdinir are pivotal in treating various infectious diseases. MCs such as magnesium oxide, ferrous citrate, and sucralfate are frequently used as antacids and cathartics. When AMAs are taken simultaneously with MCs, they chelate, reducing their absorption and therapeutic efficacies [6–8]. Despite the well-known risks, the prescriptions that order co-intake of AMAs and MCs are still seen in clinical practice. While pharmacists have often been alerted to this risky interaction, it is not sufficient to reduce those prescriptions.

Clinical Decision Support Systems (CDSSs) have emerged as powerful tools in the healthcare landscape, significantly enhancing the ability to prevent adverse drug interactions. These systems utilize comprehensive databases and sophisticated algorithms to provide real-time alerts to healthcare providers about potential drug-drug interactions, thereby preventing patient harm. Numerous studies have demonstrated the efficacy of CDSSs in improving medication safety and optimizing therapeutic outcomes. For instance, research has shown that the implementation of CDSSs can reduce the incidence of serious drug-drug interactions and associated adverse events by alerting clinicians to potential interactions at the point of care [9–11]. In this study, we focused on the interaction between AMAs and MCs to facilitate the appropriate use of AMAs.

### Main text

**Fig. 1:**
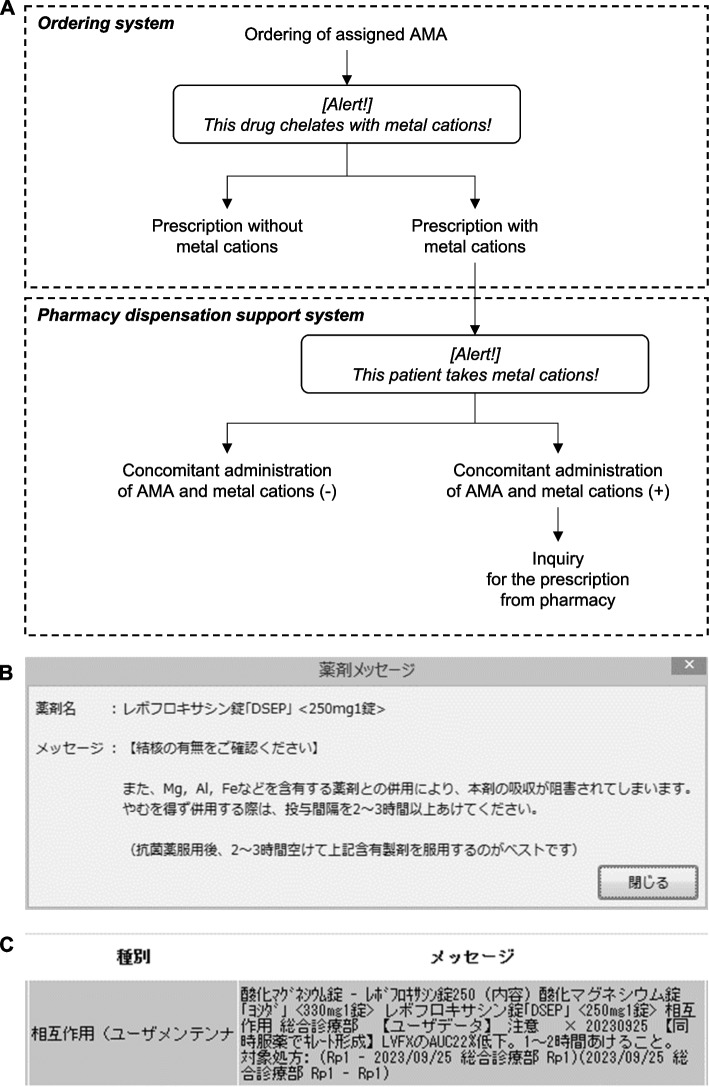
Automated pop-up alerts implemented in our ordering and pharmacy dispensation support system. **A** The flow of pop-up alerts in our ordering and pharmacy dispensation support system. **B**, **C**)The displays of pop-up alerts on ordering (B) and pharmacy dispensation support system (C)

We investigated the effectiveness of pop-up alerts integrated into our hospital’s ordering and pharmacy dispensation support systems in reducing the prescriptions that ordered co-intake of AMAs and MCs. To this end, we implemented our ordering system HAPPY ACTIS (CANON MEDICAL SYSTEMS CORPORATION, Tochigi, Japan) to pop up when the assigned AMAs (Quinolones, Tetracyclines, and Cefdinir) were ordered, regardless of whether or not the prescriptions contained MCs (Fig. 1A, B). We also implemented our hospital’s pharmacy dispensation support system YUNICOM-EX (Yuyama MFG Co., Ltd., Osaka, Japan) to pop up when the assigned AMAs or MCs were prescribed for the patient who was on MCs or the assigned AMAs, respectively (Fig. 1A, C). The detailed settings for pop-up alerts in the ordering and pharmacy dispensation support systems were shown in the Supplemental Materials. We then surveyed the number of prescriptions ordering co-intake of AMAs and MCs and the number of pharmacist inquiries to prevent those prescriptions between Feb 2016 – Jan 2017 (Pre), Feb 2017 – Jan 2018 (Post 1y), and Feb 2018 – Jan 2019 (Post 2y). This study was approved by the St. Mary’s Hospital Institutional Review Board (approval number: 19–1006). All statistical analyses were performed using EZR software [12]. Proportions were compared using Chi-square test or Fisher's exact test for every pair, and P values due to repeated multiple testing were corrected using the Bonferroni method. *P* value < 0.05 was regarded as statistically significant.

**Fig. 2:**
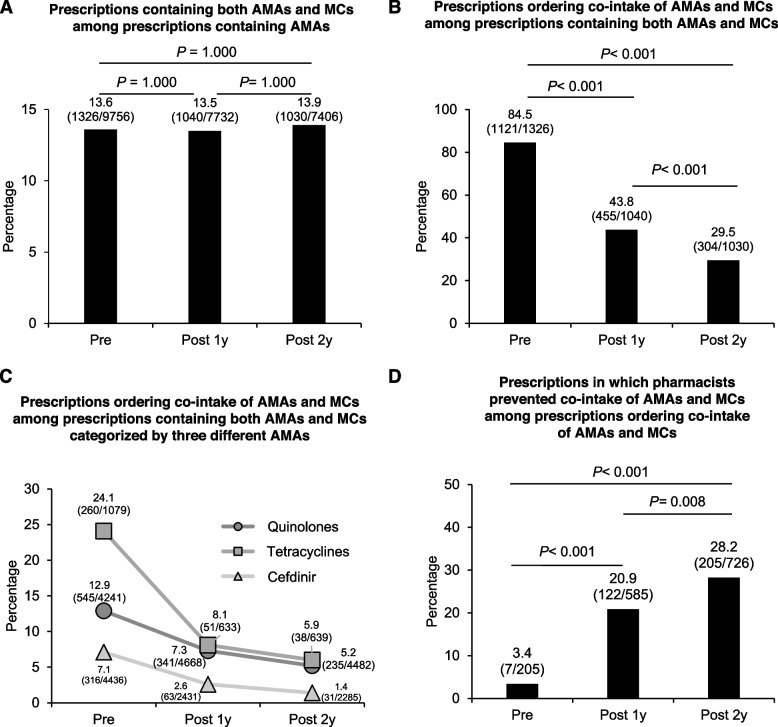
The effectiveness of the implemented automated pop-up alerts in reducing the prescriptions ordering co-intake of AMAs and MCs. **A-D** The proportion and number of prescriptions containing both AMAs and MCs among prescriptions containing AMAs (A), prescriptions ordering co-intake of AMAs and MCs among prescriptions containing both AMAs and MCs (B), prescriptions ordering co-intake of AMAs and MCs categorized by Quinolones, Tetracyclines and Cefdinir (C), prescriptions in which pharmacists prevented co-intake of AMAs and MCs among prescriptions ordering co-intake of AMAs and MCs (D), before and after implementation of the automated pop-up alerts. The difference in significance of the ratio was analyzed by the Chi-square test (for A, B) and Fisher’s exact test (for C, D), followed by the Bonferroni method. P value < 0.05 was regarded as statistically significant

We first examined the effect of the pop-up alerts on the number of prescriptions ordering co-intake of AMAs and MCs during Pre, Post 1y, and Post 2y. There was no difference in the percentage of prescriptions including AMAs and MCs between before and after the introduction of pop-up alerts, indicating that the pop-up alerts did not abolish physicians to order AMAs (Fig. 2A). On the contrary, the proportion of prescriptions ordering co-intake of AMAs and MCs was significantly decreased after the introduction of pop-up alerts as compared with before the implementation of pop-up alerts (Fig. 2B). Of note, the proportion of prescriptions ordering co-intake of AMAs and MCs was significantly lower in the Post 2y than Post 1y (Fig. 2B). We further analyzed the trend in the proportion of prescriptions ordering co-intake of AMAs and MCs, categorized by type of AMAs. The proportion of prescriptions ordering co-intake of Quinolones, Tetracyclines, or Cefdinir and MCs was significantly reduced respectively (Fig. 2C). Of note, the ratio of prescriptions ordering co-intake of tetracyclines and MCs was dramatically decreased (Fig. 2C). In our hospital, Tetracyclines are frequently used for osteomyelitis, skin and soft tissue infection, and molecular targeted therapy-induced skin disorders. The reduction of prescriptions ordering co-intake of AMAs and MCs is thought to reflect the usefulness of the induction of pop-up alerts in our hospital’s ordering system.

We implemented the ordering system to alert for all cases when the physicians ordered the assigned AMAs, regardless of whether the physicians ordered the MCs. On the other hand, we did not implement the ordering system to notify whether other physicians had ordered the MCs before. Therefore, we also implemented the pharmacy dispensation support system to alert when the MCs were included in the prescription of the specific patient (Fig. 1A). If the pharmacists find the prescriptions ordering co-intake of AMAs and MCs, they can inquire about preventing the co-intake of AMAs and MCs to the physicians. We examined the proportion of prescriptions in which pharmacist inquiries prevented the co-intake of AMAs and MCs among prescriptions ordering co-intake of AMAs and MCs. The proportion of prescriptions in which pharmacist inquiries prevented the co-intake of AMAs and MCs significantly increased after the implementation of pop-up alerts compared to before the implementation of pop-up alerts in the pharmacy dispensation support system. Of note, the ratio of prescription inquiry for co-intake of AMA and MCs was significantly higher in Post 2y than in Post 1y (Fig. 2D). This result indicates that pop-up alerts in pharmacy dispensation support system promoted the pharmacist’s activity for preventing the co-intake of AMAs and MCs.

### Limitations of this study

In recent years, CDSS has significantly contributed to reducing adverse drug interactions through automated alerts. On the contrary, previous studies have demonstrated that excessive alerts can overwhelm physicians, overriding a substantial proportion of alerts including those that could prevent serious drug interactions [13–15]. Indeed, although our pop-up alerts on the ordering system significantly reduced the prescription ordering co-intake of AMAs and MCs, many of those prescriptions are still seen, indicating that pop-up alerts were often overridden. A prolonged study is required to fully understand our dual alert system’s long-term effects. Additionally, external validity is lacking because this study was conducted in only one hospital. A multicenter study is required to evaluate the reproducibility and usefulness of our dual alert system.

## Conclusions

In this study, we investigated the effects of pop-up alerts in the ordering system and the pharmacy dispensation support system on reducing the prescriptions that order the co-intake of AMAs and MCs. We found that [1] pop-up alerts in the ordering system significantly reduced the prescriptions that ordered co-intake of AMAs and MCs, and [2] pop-up alerts in the pharmacy dispensation support system increased pharmacist inquiries for the prescriptions that ordered the co-intake of AMAs and MCs. We found both pop-up alerts helped reduce those risky prescriptions. This result suggests that improving physician’s awareness and knowledge can directly change prescribing behaviors. Furthermore, the increase in the number of inquiries from pharmacists due to the pop-up alerts in the dispensing support system reaffirms the crucial role of pharmacists. This outcome indicates that appropriate information provision contributes to high-quality medical care for patient safety. The findings of this study have clarified that the utilization of information technology can effectively enhance the quality of medical care. On the contrary, several issues remain. Our ordering system has implemented many pop-up alerts besides pop-up alerts for the prescriptions that order co-intake of AMAs and MCs. It has been reported that the physician’s attention was gradually decreased in a time-dependent manner, and implementation of computerized provider order entry often led to unintended consequences and alert fatigue, which limit the system's safety effects [16]. Therefore, several issues require further research, such as optimizing pop-up frequency and content and educating and training physicians and pharmacists. In summary, this research has highlighted the effects and significance of information provision in the medical field. Moving forward, it will be essential to undertake initiatives based on these insights to ensure the delivery of even higher-quality medical care.

## Supplementary Information

Supplementary Material 1.

## Data Availability

The data that support the finding of this study are available from the corresponding author.

## Abbreviations

AMAs: Antimicrobial agents
MCs: Metal cations
