# Supplementary material for: Impact of automated pop-up alerts on simultaneous prescriptions of antimicrobial agents and metal cations
Source: J Pharm Health Care Sci. 2024 Sep 27;10:59. doi: 10.1186/s40780-024-00377-3 (PMC11430289; doi:10.1186/s40780-024-00377-3)

**Ⅰ. Implementation of pop-up alert in the ordering system HAPPY ACTIS**

1. Write a message that will pop up when the physicians explore the drug. Then, assign the message key for the message in the message node (drug exploring) of the ‘prescription order maintenance—comments for drugs’ function.


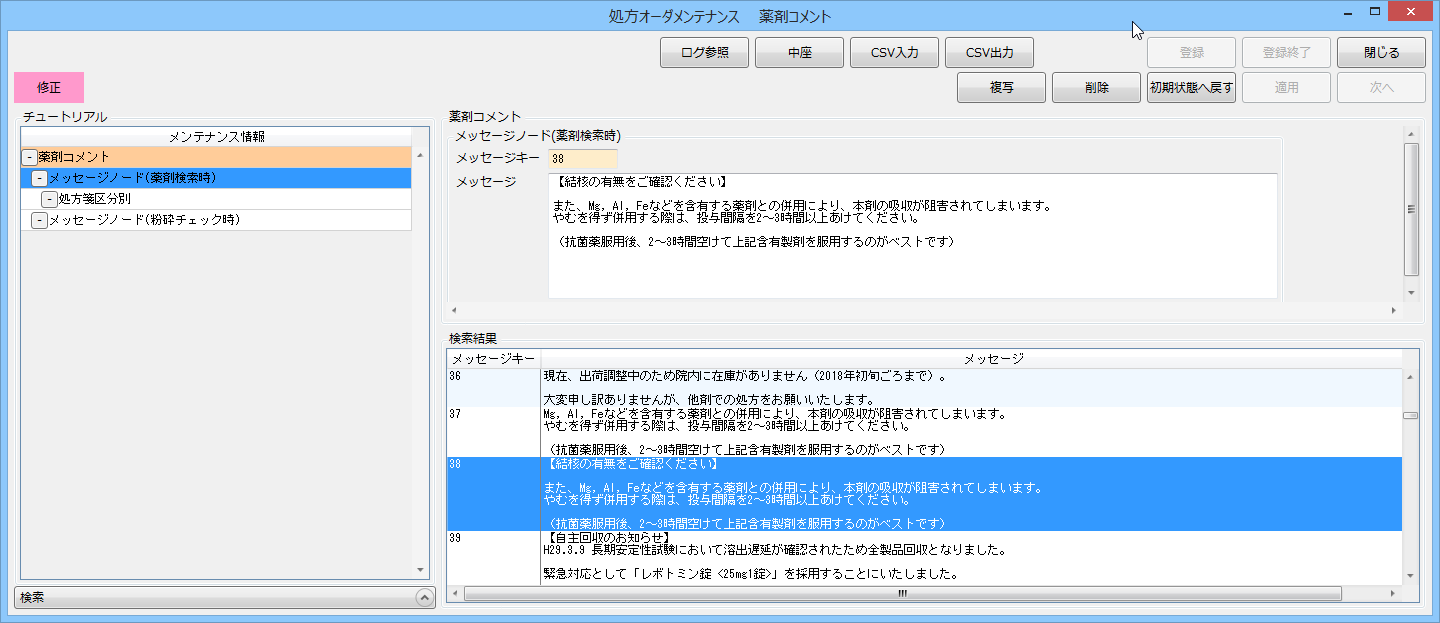


2. The messages and key were also in the ‘prescription order maintenance—change or cancel of the drugs’ function.


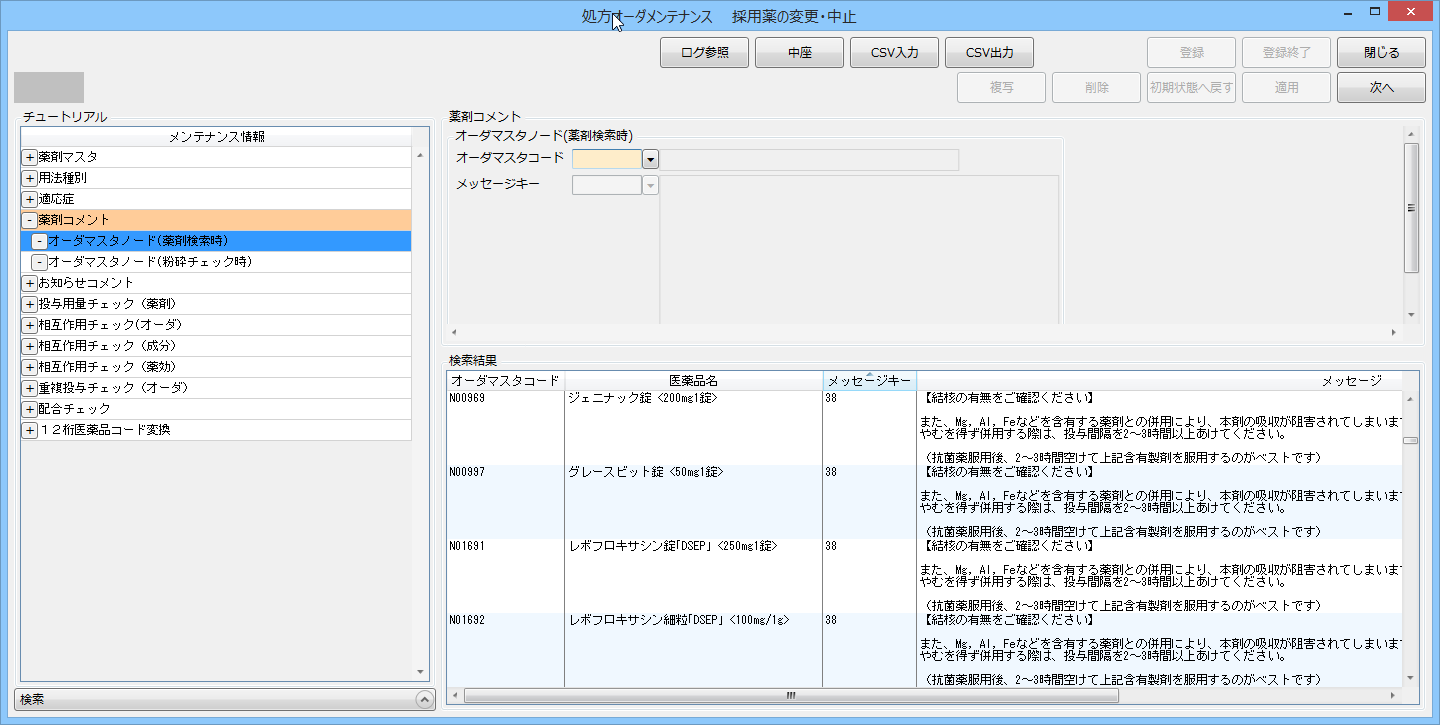


These settings pop up the message when the assigned AMAs are prescribed, regardless of whether or not the prescription includes MCs.

**Ⅱ. Implementation of pop-up alert in the dispensation support system YUNICOM-EX**

1. Open the ‘drug-drug interaction master’ function.


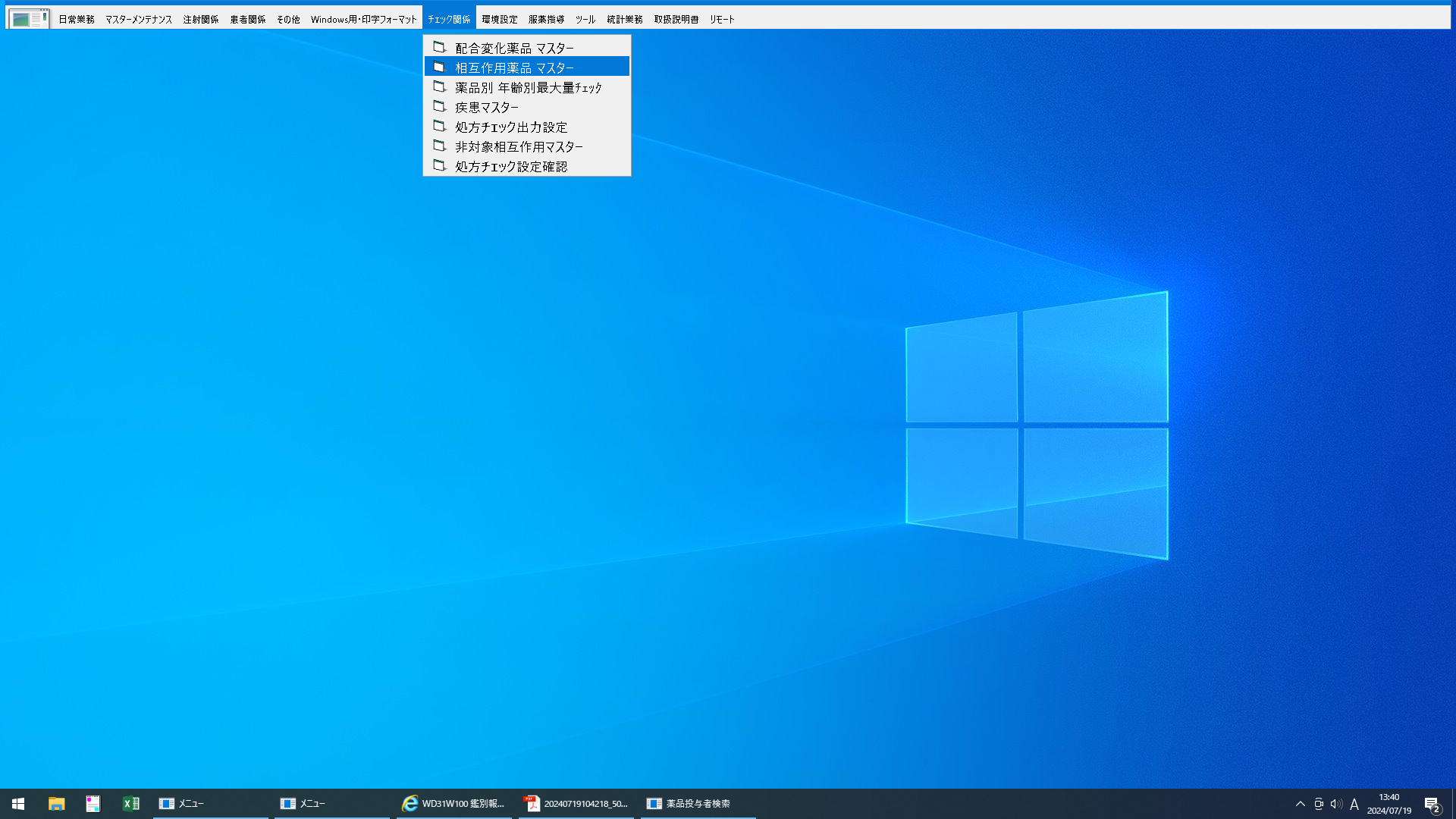


2. First, we assigned the drug code to the AMAs. Next, we linked the AMAs and the MCs that could chelate with the target AMAs in the 'drug-drug interaction master maintenance' function. This setting pops up a message in the dispensation support system when the prescription includes the assigned AMAs and MCs. The messages were changed for each AMA because the absorption reduction rate varies depending on the combination of AMA and MC.


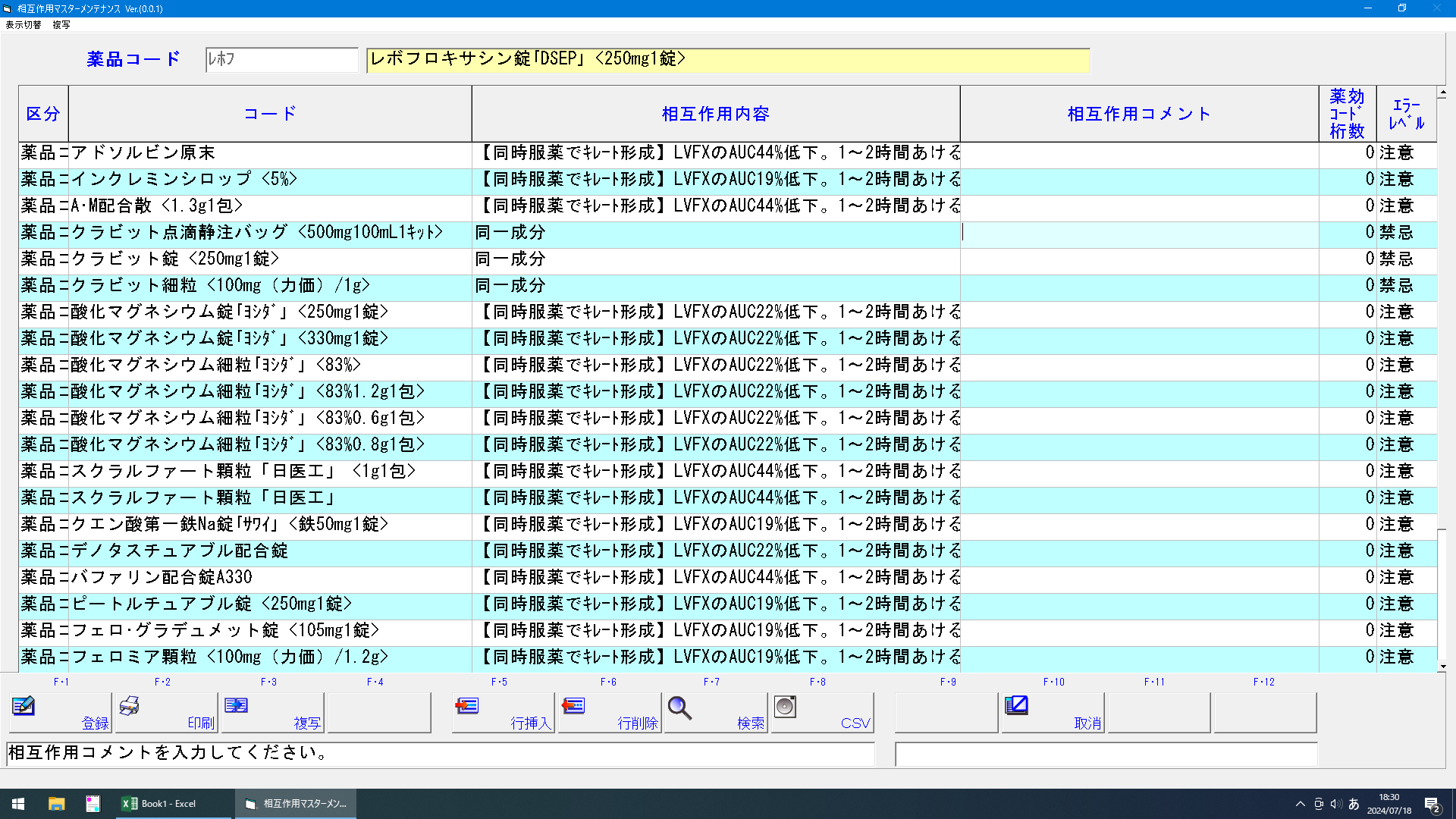


3. Additionally, we configured the 'drug-drug interaction master maintenance' function in the dispensation support system to pop up the alert in the three cases below.

1) AMAs were prescribed when the patient was on MCs.

2) MCs were prescribed when the patient was on AMAs.

3) AMAs and MCs were simultaneously prescribed.
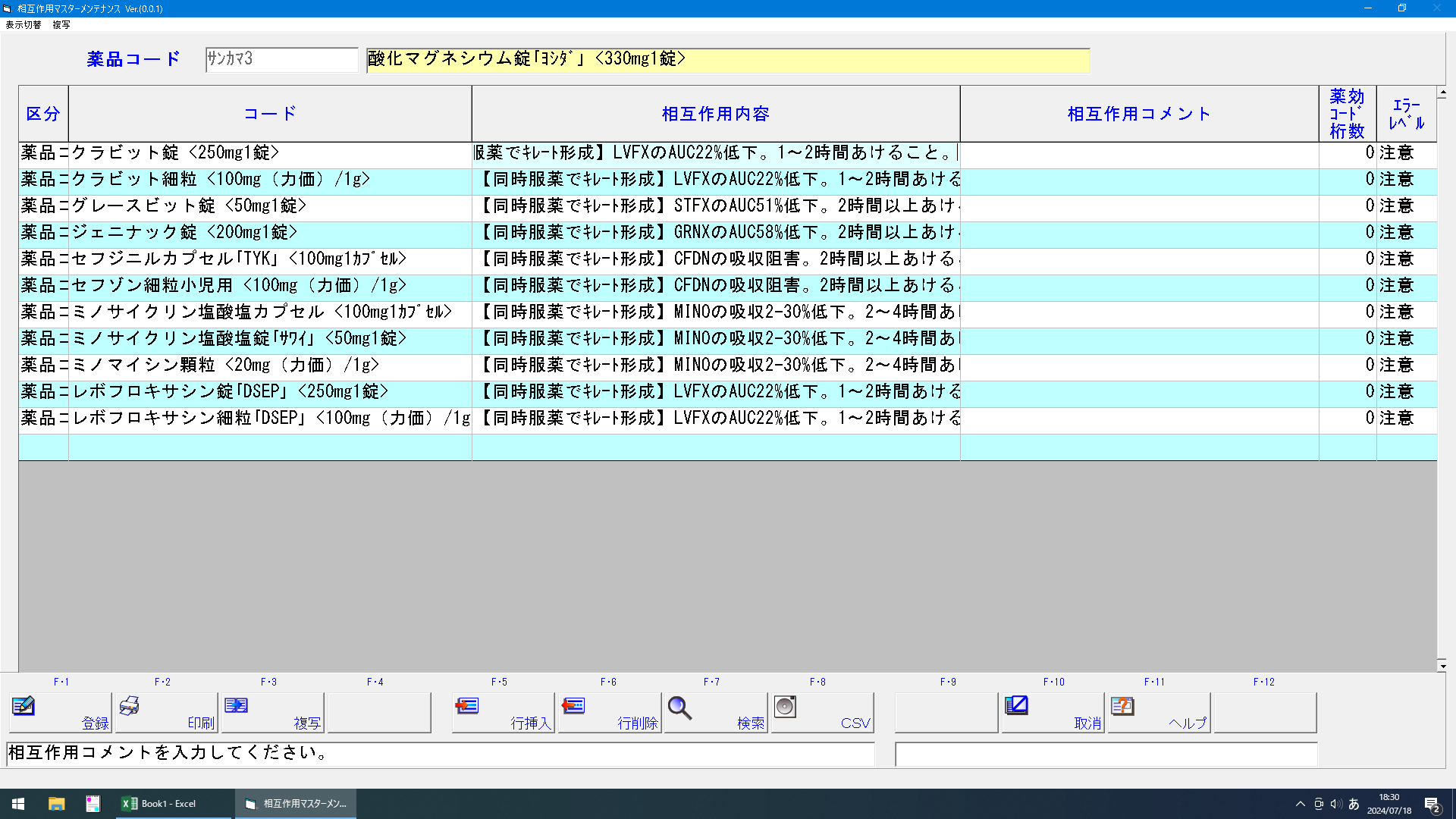

Supplement: Supplementary file 1 — Supplementary Material 1. [file 40780_2024_377_MOESM1_ESM.docx]
